# Akt-activated endothelium promotes ovarian cancer proliferation through notch activation

**DOI:** 10.1186/s12967-019-1942-z

**Published:** 2019-06-10

**Authors:** Jessica Hoarau-Véchot, Cyril Touboul, Najeeb Halabi, Morgane Blot-Dupin, Raphael Lis, Charbel Abi Khalil, Shahin Rafii, Arash Rafii, Jennifer Pasquier

**Affiliations:** 1 Stem Cell and Microenvironment Laboratory, Weill Cornell Medicine-Qatar, Education City, Qatar Foundation, Doha, Qatar; 2 Epigenetics Cardiovascular Laboratory, Department of Genetic Medicine, Weill Cornell Medicine-Qatar, PO box 24144, Doha, Qatar; 3 INSERM U955, Equipe 7, Créteil, France; 4 Faculté de Médecine de Créteil UPEC–Paris XII, Service de Gynécologie-Obstétrique et Médecine de la Reproduction, Centre Hospitalier Intercommunal de Créteil, 40 Avenue de Verdun, 94000 Créteil, France; 5 Department of Genetic Medicine, Weill Cornell Medicine, New York City, NY USA; 6 Department of Gynecologic Oncology, Hospital Foch, Surresnes, France; 7 Department of Genetic Medicine and Obstetrics and Gynecology, Stem Cell and Microenvironment Laboratory, Weill Cornell Medicine-Qatar, Qatar-Foundation, PO: 24144, Doha, Qatar

**Keywords:** Ovarian cancer, Tumor microenvironment, Cell–cell interactions, Endothelial cells

## Abstract

**Background:**

One main challenge in ovarian cancer rests on the presence of a relapse and an important metastatic disease, despite extensive surgical debulking and chemotherapy. The difficulty in containing metastatic cancer is partly due to the heterotypic interaction of tumor and its microenvironment. In this context, evidence suggests that endothelial cells (EC) play an important role in ovarian tumor growth and chemoresistance. Here, we studied the role of tumor endothelium on ovarian cancer cells (OCCs).

**Methods:**

We evaluated the effect of activated endothelial cells on ovarian cancer cell proliferation and resistance to chemotherapy and investigated the survival pathways activated by endothelial co-culture.

**Results:**

The co-culture between OCCs and E4^+^ECs, induced an increase of OCCs proliferation both in vitro and in vivo. This co-culture induced an increase of Notch receptors expression on OCC surface and an increase of Jagged 1 expression on E4^+^ECs surface and activation of survival pathways leading to chemoresistance by E4^+^ECs.

**Conclusion:**

The targeting of aberrant NOTCH signaling could constitute a strategy to disrupt the pro-tumoral endothelial niche.

## Background

The concept of cancer as a cell-autonomous disease has been challenged by the wealth of knowledge gathered in the past decades on the importance of tumor microenvironment (TM) in cancer progression and metastasis [1, 2]. The significance of endothelial cells (ECs) in this scenario was initially attributed to their role in neo-angiogenesis that in fact is critical for tumor initiation and growth. Nevertheless, the identification of endothelial-derived angiocrine factors illustrated an alternative non-angiogenic function of ECs contributing to both physiological and pathological tissue development. Gene expression has demonstrated different patterns of expression in endothelial cells extracted from tumor, implying bilateral cross-talk [3]. ECs from tumor display a proangiogenic phenotype regulated by the activation of the phosphatidylinositol 3-kinase/Akt pathway [4]. Our team, among others, showed that cancer cells were able to activate Akt pathway in normal endothelium [5–7]. We also demonstrated that Akt-activated ECs provide a notch-dependent pro-tumoral niche enhancing breast cancer survival, stemness and pro-metastatic properties [8].

Although classically known for its role in embryonic development, the Notch pathway is now being recognized for its deregulation in cancer [5, 8, 9]. Within all four Notch receptors, Notch3 is amplified in ovarian cancer and associated with its progression [10]. Activation of Notch3 dependent pathway in ovarian cancer regulates ovarian cancer cells (OCC) adhesion to peritoneal cells and cancer cell metastatic outgrowth [11]. Patients with high grade serous ovarian adenocarcinomas showing high Notch3 expression have a significantly worse clinical outcome, including reduced overall survival and shortened progression-free survival than patients with low Notch3 expression [12].

In this study, we aimed to explore the role of tumor ECs on OCC. To model tumor endothelium, we used our model of Akt-activated endothelial cells (E4^+^ECs). We demonstrated that Notch3 activation by Endothelial-Jagged1 leads to increased proliferation and chemoresistance in OCC.

## Methods

### Cell cultures

Ovarian cancer cell lines Skov3 were purchased from ATCC and cultured following ATCC recommendations (ATCC, Manassas, VA, USA). A primary ovarian cancer cell line was derived in our laboratory from ascites of a patient with Stage III serous adenocarcinoma (APOCC). The cell lines were cultured in DMEM high glucose (Hyclone, Thermo Scientific), 10% FBS (Hyclone, Thermo Scientific), 1% Penicillin–Streptomycin-Amphotericyn B solution (Sigma), 1X Non-Essential Amino-Acid (Hyclone, Thermo Scientific) and 1% l-glutamine. Cultures were incubated in humidified 5% CO2 incubators at 37 °C and the media was replaced every 3 days.

We used our model of HUVECs with autonomous Akt-activation surviving in the absence of FBS and cytokines (ECs) as a surrogate for tumor-associated endothelium [5–7, 13]. E4orf1 transfected HUVEC (E4 + EC) were obtained as previously described [14]. HUVECs were purchased from ATCC and cultured following ATCC recommendations (ATCC, Manassas, VA, USA). Cells were cultured in endothelial cell growth medium (Medium 199, 20% (v/v) fetal bovine serum (FBS), 20 μg ml–1 endothelial cell growth supplement (Hallway), 1% (v/v) antibiotics (Hallway), and 20 units ml–1 heparin). In the E4 + EC model the transfection of the adenoviral cassette E4orf1 in HUVECs provides low level of Akt activation allowing the use of serum-free, cytokine-free media without inducing immortalization nor altering the endothelial phenotype [14].

### Xenograft study

All animal procedures were approved by the institutional animal care and use committee (IACUC) of Weill Cornell Medical College. For OCC xenografts, 26105 Skov3 cells were injected solely, or in 1:10 mixture with either 26106 E4 + ECs, subcutaneously into NOD.Cg-Prkdcscid Il2rgtm1Wjl/SzJ (NSG) recipient mice. Seven weeks post xenograft injection, tumors were isolated for quantification and imaging. Isolated tumors were immediately embedded in Tissue-Tek embedding media (Sakura, 4583) and were then snap frozen in liquid nitrogen. Consequently, 5-mm sections were prepared and stained with PE-CD31 antibody (BD Biosciences, 560983) to check blood vessel density. Images were taken from tumor foci with Nikon Eclipse TE 2000-U.

### Cell proliferation assay

Cells were plated at 50,000 cells per well in a 6 well plate in medium without FBS. Cells were then counted with a hemocytometer for the following 6 days every 2 days. Two wells were counted per condition. The experiment was performed in triplicate.

### SiRNA treatment

siRNA against human Jagged1 (Santa Cruz biotechnology) were introduced into cells by lipid mediated transfection using siRNA transfection medium, reagent and duplex (Santa Cruz biotechnology) following manufacturer recommendations. Briefly the day before transfection cells were plated at 2, 5·10^5^ cells per well in 2 ml antibiotic-free normal growth medium supplemented with FBS. Cells were incubated until they reach 60–80% confluence. The duplex solution containing the siRNA is then added to the cells. After 5 to 7 h, antibiotics are added in each well and the cells are incubated for 24 h more. The media is then replaced by normal growth media and cells are used for experiments and assay by RT-PCR to analyze the expression of Jagged1 gene.

### shRNA transfection

Lentiviral particles containing shRNA against human Jagged1 (sc-37202-V), scrambled lentiviral particles (sc-108080), and polybrene (sc-134220) were purchased from Santa Cruz Biotechnology (USA). In summary, E4-ECs were cultured up to 50% confluence and were then treated with polybrene and lentiviral particles containing shRNA against Jagged1 or scrambled particles. Transfected cells were then selected using puromycin (Sigma, USA), and down-regulation of Jagged1 was assessed by qPCR [6].

### Flow cytometry

Fluorescence (FL) was quantified on a SORP FACSAria2 (BD Biosciences) as previously described [13, 15]. Data were processed with FACSDiva 6.3 software (BD Biosciences). Doublets were excluded by FSC-W x FSC-H and SSC-W x SSC-H analysis. eGFP fluorescence was acquired with 488 nm blue laser and 510/50 nm emission, EpCam APC conjugated (BD Biosciences) was acquired with 647 nm red laser and 670/14 nm emission, Pkh red fluorescence was acquired with 535 nm green laser and 582/15 nm emission. Charts display the median of fluorescence intensity (mfi) relative to control. Single stained channels were used for compensation and fluorophore minus one (FMO) controls were used for gating. 20,000 events were acquired per sample. Viability was assessed by flow cytometry evaluation of Calcein AM staining as described by the manufacturer (Live Dead Viability/Cytotoxicity Kit, Molecular Probes, Invitrogen).

### Confocal microscopy

The interactions between PKH26^+^OCCs and GFP^+^E4 + ECs in angiosphere were imaged using a Zeiss confocal Laser Scanning Microscope 710 (Carl Zeiss). Post-acquisition image analysis was performed with Zeiss LSM Image Browser Version 4.2.0.121 (Carl Zeiss). Spheres were imaged live using glass bottom microwell plates (MatTek Corporation, USA).

### Western blots analysis

Western blots were carried out as previously described [16]. Immunostaining was carried out using a goat monoclonal antibodies against phospho-Akt (S473) (Cell Signaling #9271) and actin (1/1000, Cell signaling) and a secondary polyclonal mouse anti-goat antibody HRP conjugated (1/2000, cell signaling). Blots were developed using HRP and chemiluminescent peroxidase substrate (#CPS1120, Sigma). Data were collected using Geliance CCD camera (Perkin Elmer), and analyzed using ImageJ software (NIH).

### RNA sequencing, gene expression and pathway analysis

RNA was extracted using RNeasy mini kit Qiagen kit and all RNA quality was assessed using the Agilent RNA 6000 Nano kit on the Agilent 2100 Bioanalyzer. Paired-end sequencing was performed on Illumina Hiseq 2500 after library creation using the Nugen Ovation Single Cell kit. Raw reads were deduplicated and aligned with RNA STAR in 2-pass mode to HG19 using GENCODE annotations. Gene read counts were obtained with Rsubread using gencode v.19 annotation and passed to EdgeR for differential expression analysis. Significant differentially expressed genes were those that with an FDR less than 0.05. Significant differentially expressed genes were uploaded to Reactome (Oct. 16, 2018) using no interactors. Significant pathways were those with an FDR less than 0.05.

### Statistical analysis

All quantitative data were expressed as mean ± standard error of the mean (SEM). Statistical analysis was performed by using SigmaPlot 11 (Systat Software Inc., Chicago, IL). A Shapiro–Wilk normality test, with a p = 0.05 rejection value, was used to test normal distribution of data prior further analysis. All pairwise multiple comparisons were performed by one way ANOVA followed by Holm–Sidak posthoc tests for data with normal distribution or by Kruskal–Wallis analysis of variance on ranks followed by Tukey posthoc tests, in case of failed normality test. Paired comparisons were performed by Student’s t-tests or by Mann–Whitney rank sum tests in case of unequal variance or failed normality test. Statistical significance was accepted for p < 0.05 (*), p < 0.01 (**) or p < 0.001 (***). All experiments were performed in triplicates.

## Results

### E4^+^ECs promote OCCs proliferation and survival properties in vitro and in vivo

**Fig. 1:**
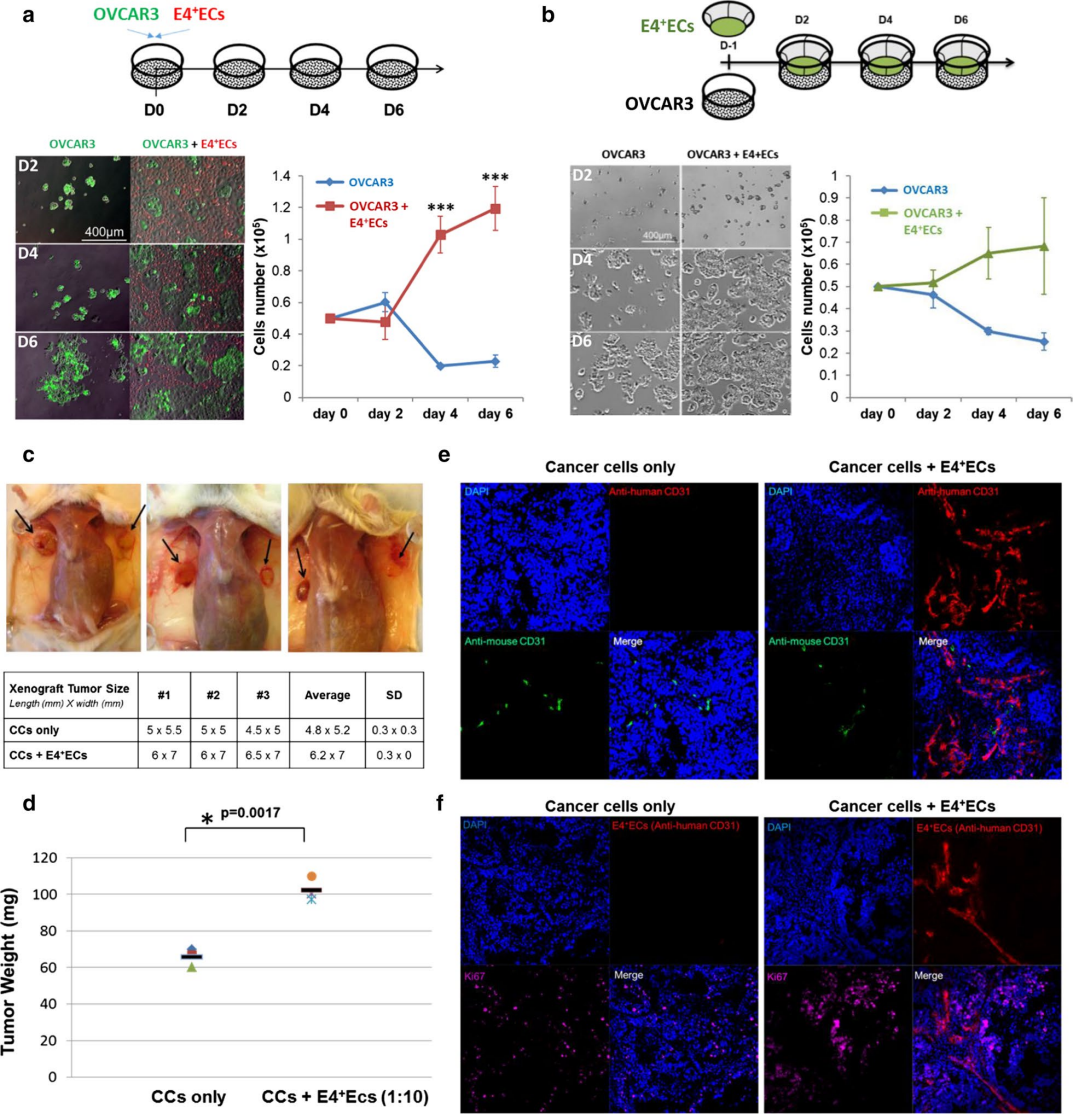
**a** Proliferation assay. OVCAR3 were plated and counted every 2 days in presence or not of E4^+^ECs for 6 days. **b** Proliferation assay in transwell. OVCAR3 were grown in a Transwell system and counted every 2 days in presence or not of E4^+^ECs for 6 days. **c** Representative pictures of the ovarian tumor seen after sacrifice of the mice. The table displays the xenograft tumor size for each mouse in millimeters (length × width). **d** The graph represents the tumor weight for each mouse in both groups. **e** Tumors were snap-frozen after isolation and then sectioned to 10 µm for immuno-staining. Slides were stained with anti-human CD31 and anti-mouse CD31 antibodies, DAPI and Immunofluorescence images were acquired in confocal microscopy. **f** Tumors were snap-frozen after isolation and then sectioned to 10 µm for immuno-staining. Slides were stained with anti-human CD31 and anti-human Ki67 antibodies, DAPI and Immunofluorescence images were acquired in confocal microscopy

In order to investigate the interaction of endothelial and ovarian cancer cells, we used E4^+^ECs as a surrogate for tumor ECs [6]. We first investigated if E4^+^ECs could provide a proliferative niche for OCCs under complete starvation using OVCAR3 cell lines in co-culture with E4^+^ECs in a serum and cytokine-free (Fig. 1a). We showed that OVCAR3 gained a pro-survival advantage through contact with E4^+^ECs. OVCAR3 showed more than a fivefold increase in their proliferation capacity 6 days post-contact with E4^+^ECs. In contrast, when OVCAR3 and E4^+^ECs were grown in a Transwell system, the proliferative advantage couldn’t be recapitulated (Fig. 1b).

We then evaluated, in vivo, the impact of E4^+^ECs in ovarian tumor growth. In a xenograft assay, we showed that co-injection of OVCAR3 with E4^+^ECs into NSG mice significantly increased tumor size (Fig. 1c) and weight (Fig. 1d). Confocal microscopy confirmed the constitution of large endothelial networks among the tumor xenograft supporting the role of the contact between cancer and endothelial cells as shown by (Fig. 1e) tumor cells ki67 staining close to the E4^+^ECs vessels (Fig. 1f).

### E4^+^ECs induce OCC growth and pro-metastatic properties through Notch activation

**Fig. 2:**
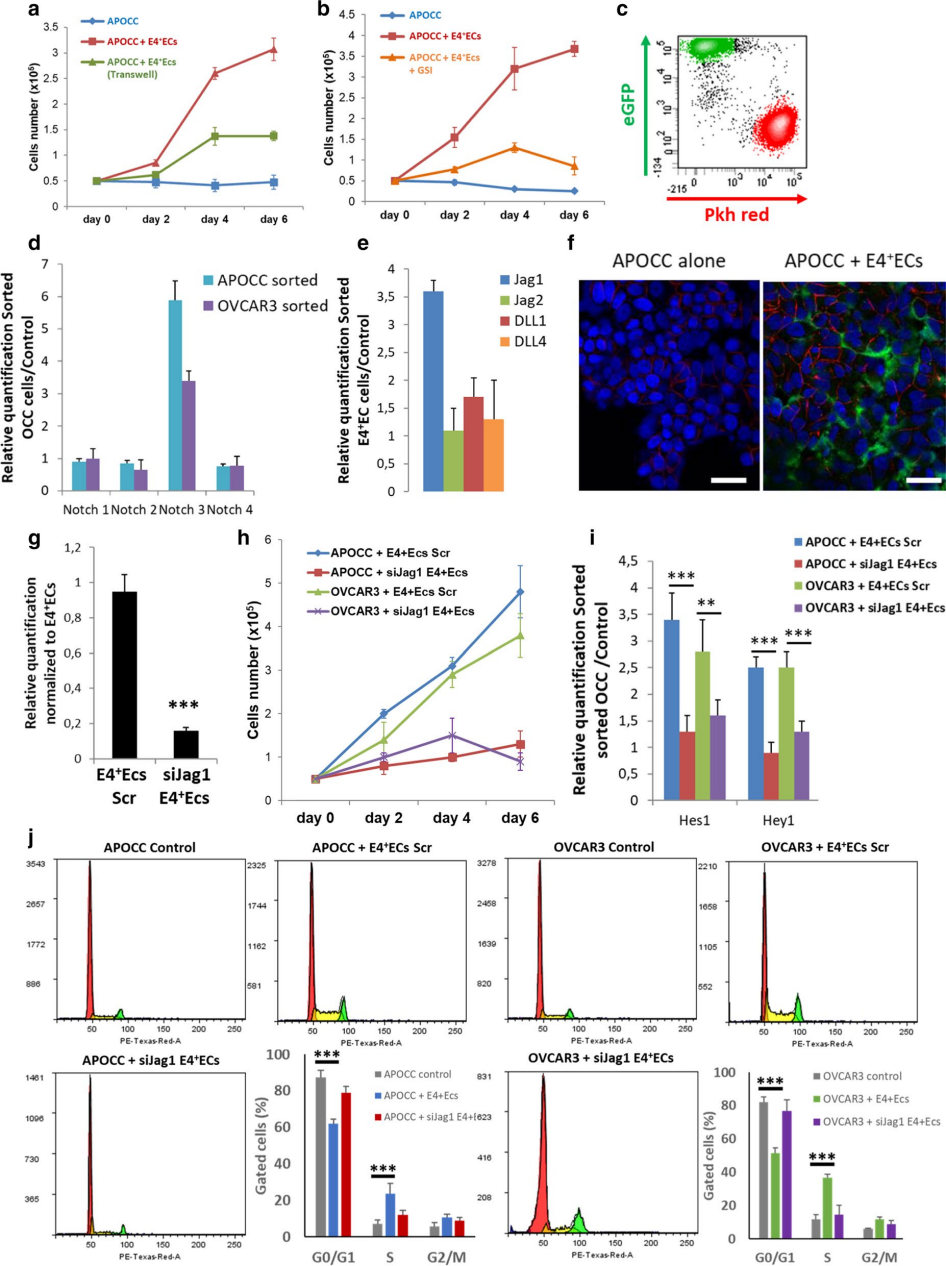
**a** Proliferation assay. APOCC were plated and counted every 2 days: alone, in coculture with E4^+^ECs or in presence of E4^+^ECs via transwell for 6 days. **b** Proliferation assay. APOCC were plated and counted every 2 days: alone, in coculture with E4^+^ECs or in coculture with E4^+^ECs with or without GSI for 6 days. **c** Flow cytometry cell sorting chart. APOCC were stained with Pkh red and were cocultured with eGFP E4^+^ECs for 2 days. E4^+^ECs (green) and OCCs (red) were gated through eGFP fluorescence intensity and Pkh red staining. **d** Real-time qPCR. APOCC and OVCAR3 were cocultured with E4^+^ECs for 2 days and sorted. The relative quantification of four Notch receptors was performed on APOCC and OVCAR3. **e** Real-time qPCR. APOCC and OVCAR3 were cocultured with E4^+^ECs for 2 days and sorted. The relative quantification of notch ligands Jagged1 (Jag1), Jagged2 (Jag2), DLL1, and DLL4 was performed on E4^+^ECs. **f** Quantification of Notch 3 expression. The upregulation of Notch3 by OCCs after coculture with E4^+^ECs was confirmed by Confocal microscopy. Scale bar 50 µm. **g** Real-time qPCR. Jag1 was quantified on E4^+^ECs scrambled or E4^+^ECs silenced for Jag1 (siJag1 E4^+^ECs). **h** Proliferation assay. APOCC and OVCAR3 were cocultured with scrambled E4^+^ECs or siJag1E4^+^ECs and counted every 2 days for 6 days. **i** Real-time qPCR. APOCC and OVCAR3 were cocultured with scrambled E4^+^ECs or siJag1E4^+^ECs and sorted. Notch downstream effectors Hes1 and Hey1 expression was assessed. **j** Cell cycle analysis. APOCC and OVCAR3 were cultured alone or cocultured with E4^+^ECs or siJag1E4^+^ECs for 2 days. Cell cycle analysis was performed by flow cytometry

We used a primary ovarian cell line derived in our laboratory from ascites of a patient with Stage III serous adenocarcinoma (APOCC). We confirmed the results observed previously for proliferation in direct co-culture or with Transwell using APOCC (Fig. 2a). To investigate the role of Notch/Jagged pathway in this phenomenon, we used a gamma secretase inhibitor (GSI) during co-culture between APOCC and E4^+^ECs (Fig. 2b). GSI inhibited the impact of E4^+^ECs on APOCC proliferation confirming the role of the Notch pathway. After 2 days of co-culture, we determined the expression of all four Notch receptors on sorted OCCs and notch ligands (Jagged1 (Jag1), Jagged2 (Jag2), DLL1, and DLL4) on sorted E4^+^ECs (Fig. 2c). qPCR analysis showed significant up-regulation of Notch 3 receptor on OCCs (Fig. 2d) and Jag1 ligand on E4^+^ECs (Fig. 2e). We confirmed the up-regulation of Notch3 on OCCs during co-culture with E4^+^ECs by confocal microscopy (Fig. 2f). To further study the role of Notch3 and its ligand Jag1 in EC-tumor interaction, we silenced Jag1 expression on E4^+^ECs using siRNA (Fig. 2g) and showed an inhibition of APOCC or OVCAR3 proliferation in co-cultures with E4^+^ECs^siJag1^ (Fig. 2h). We sorted the OCCs and showed a decrease in the expression of Notch downstream effectors Hes1 and Hey1 (Fig. 2i). Finally, we looked at the cell cycle in flow cytometry and showed a decrease of the G0/G1 population and an increase of S population in OCC during co-culture with E4^+^ECs^siJag1^ (Fig. 2j).

### E4^+^EC activation of Notch pathway in OCC mediates chemoresistance

**Fig. 3:**
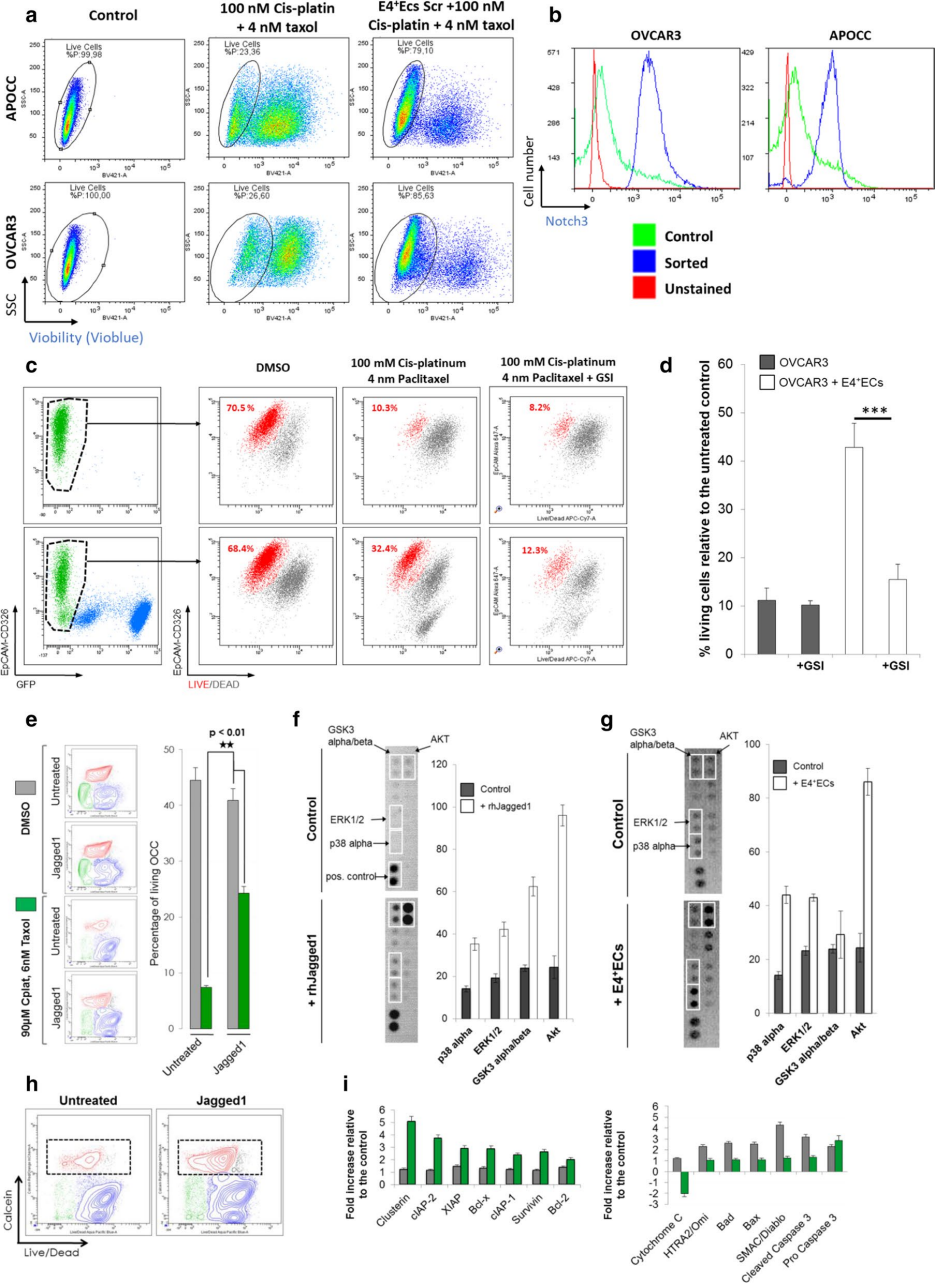
**a** Flow cytometry viability assay. APOCC and OVCAR3 were treated or not with 100 nM cisplatin and 4 nM taxol alone or in presence of E4^+^ECs for 48 h. Viability was assessed by flow cytometry using Vioblue. **b** Notch expression quantification. APOCC and OVCAR3 were treated or not with 100 nM cisplatin and 4 nM taxol. Surviving APOCC and OVCAR3 were sorted and Notch3 expression was quantified by flow cytometry. **c**, **d** Viability assay. OCCs in coculture with E4^+^ECs were treated either with 100 nM cisplatin and 4 nM taxol, or with 100 nM cisplatin, 4 nM taxol and GSI; Control: OCCs cocultured with E4^+^ECs in DMSO. Cells were stained with live/dead and live cells (in red) were quantified by flow cytometry. The histogram displays the percentage of living cells for each condition. **e** Viability assay. OVCAR3 were treated with 100 nM cisplatin and 4 nM taxol after treatment with human recombinant (rh) Jagged1 or not. Viability was assessed by flow cytometry. **f** Phosphokinase array. OVCAR3 were treated in the presence or not of rh Jagged 1. The significant protein modifications of the phosphokinase array were cut from the full membrane and represent on the left panel. The right panel represents the relative quantification of the dot pixel density. **g** Phosphokinase array. OVCAR3 were cultured with or without E4^+^ECs and sorted. The significant protein modifications of the phosphokinase array were cut from the full membrane and represent on the left panel. The right panel represents the relative quantification of the dot pixel density. **h** Viability assay. OVCAR3 were treated with 100 nM cisplatin and 4 nM taxol after treatment with human recombinant (rh) Jagged1 or not. Surviving OVCAR3 were sorted. **i** Apoptosis array. Surviving OVCAR3 treated with 100 nM cisplatin and 4 nM taxol after upstream treatment with Jagged1 were analyzed using an apoptosis array. The histograms represent the relative quantification of the dot pixel density

One of the major issues in ovarian cancer is chemoresistance. We assessed if E4^+^ECs could mediate chemoresistance in OCC. After 48 h of chemotherapy treatment (100 nM Cis-platinium + 4 nM taxol), APOCC and OVCAR3 co-cultured with E4^+^ECs showed significant chemoresistance (Fig. 3a). We investigated the role of Notch-Jagged pathway in chemoresistance. We demonstrate an increase of Notch3 in the surviving OCCs after treatment with chemotherapy (100 nM Cis-platinium + 4 nM taxol, Fig. 3b). We then showed that GSI is able to reverse the chemoresistance induced by the E4^+^ ECs (Fig. 3c, d). Concordantly when treated with a human recombinant (rh) Jagged1, OVCAR3 exhibited resistance to chemotherapy treatment (90 nM Cis-platinium + 6 nM taxol) as induced by E4^+^ECs co-culture (Fig. 3e). Jagged1 treatment activated several phosphokinase pathways in OVCAR3 such as AKT, GSk3α/β or ERK1/2 (Fig. 3f). Using a cell sorting strategy, we demonstrated the activation of the same pathways in OVCAR3 after co-culture with E4^+^ECs (Fig. 3g). Finally, OCCs who survived after a chemotherapy treatment in presence or not of Jagged1 were cell sorted (Fig. 3h). Using a human apoptosis antibody array, we demonstrated an increase of pro-survival protein in cells treated with Jagged1 and a decrease of pro-apoptotic ones in comparison to the cells not treated with Jagged 1 (Fig. 3i).

### Gene expression analysis

**Fig. 4:**
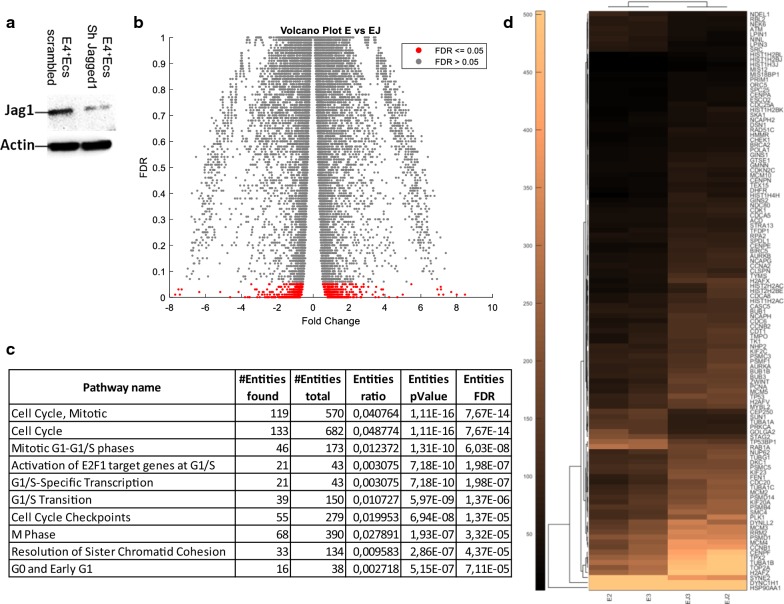
**a** Western Blot. E4 + ECs scrambled and SH for jagged 1 were done and western blot analysis was performed to confirm the silencing of jagged 1 in E4 + ECs Sh jagged 1. **b** Volcano plot showing the fold change and FDR values for all genes. Significant genes are those with an FDR values ≤ 0.05 and are indicated in red. **c** Top 10 most significant pathways identified by Reactome. **d** Hierarchical clustering of cell cycle genes merged from different pathways for each replicate of E and EJ. Values displayed are normalized CPM

To confirm the role of Notch pathway in the cross-talk between tumor and endothelium in ovarian cancer, we established a Sh-RNA for Jagged1 in E4^+^ECs (Fig. 4a) and set up long term co-culture. Briefly, we cultured APOCC alone, or in co-culture with E4^+^ECs scrambled (E) or with E4^+^ECs Sh-Jag1 (EJ) for 20 passages (endothelial cells were renewed at every passage). We then performed a transcriptomic analysis. The volcano plot in Fig. 4b is showing the fold change and FDR values for all genes. We performed a pathways analysis with Reactome (Fig. 4c) and demonstrated that all the proliferation pathways were upregulated in APOCC after a long co-culture with E4^+^ECs (E) but not after a co-culture with E4^+^ECs SH-Jag1 (EJ). The top 10 most significant pathways identified by Reactome are represented in Fig. 4c. The hierarchical clustering of cell cycle genes merged from different pathways for each replicate of E and EJ is represented in Fig. 4d, showing that the Notch pathway activation due to the contact between endothelial cells and cancer cells is maintained in long co-culture setting.

## Discussion

Here, we demonstrated using a co-culture model that activated endothelium induces increased proliferation and chemoresistance in ovarian cancer cell lines through the activation of Notch signaling. We showed that Notch receptor expression and activation is increased in co-culture and in OCCs resistant to chemotherapy suggesting this pathway to be linked to contact mediated chemoresistance.

Failure to control cancer is due to factors such as tumor heterogeneity and tumor interaction with its microenvironment [17, 18]. Indeed, the complex interplay of cancers cells with the non-cellular and cellular elements of the microenvironment are implicated in tumor growth and resistance to therapy [19–21]. Among the different component of the microenvironment, endothelial cells have been largely investigated and the ruling hypothesis is their role in constituting the vessels bringing oxygen and nutrients to the tumor. This has led to the development of many anti-angiogenic therapies that had mitigated success in clinical trials [22]. Recently our group has illustrated in many settings the angiocrine role of the endothelium, where the secreted and membrane bound factors can modulate tumor phenotype [5, 7–9, 23–25]. Other groups have clearly illustrated such perfusion independent role for endothelial cells [26–28]. Among them, Bissell group has shown the critical role of the endothelial niche in tumor dormancy and metastatic activation [28].

Most patients with ovarian cancer will undergo recurrence despite an initial good response to chemotherapy and major debulking surgery aiming at no tumor residue [18]. The particularity of ovarian cancer is the late diagnosis at an advanced stage when the tumor has invaded most of the abdominal structure including the peritoneum [29]. In these sites, tumor cells do interact with different cell types including endothelial cells. Our study illustrates how such interaction could lead to increased proliferation and resistance to therapy through a Notch pathway activation within an endothelial metastatic niche. Notch signaling is activated by a receptor-ligand binding between two cells, and signals through transcription factors such as Hes and Hey or other pathways including cyclin D1 and p21, NF-κB family members, c-Myc and Deltex [30]. The Notch pathway hence interacts with many major pro-tumoral pathways such as TGF-β, wnt signaling and GFR/HER2 receptor tyrosine kinase family, as well as phosphatidylinositol 3-kinase/AKT/mTOR signaling cascade, that are central growth pathways in both physiologic and neoplastic setting [31]. In our study, we demonstrated using gene expression the up-regulation of Notch downstream effectors following co-culture as well as the many pro-survival effectors of chemoresistance. Notch3 and Jagged2 are de-regulated in ovarian cancer, [32]. In the TCGA study, Notch signaling is altered in 22% of the patients (usually through amplification) with Notch3 alterations in 50% of those cases [33]. Among Notch ligands, Jagged1 was shown to be the most highly expressed ligand in ovarian cancer cells and surrounding peritoneal mesothelial cells [34]. Here, we showed that Jagged 1 was also the most expressed ligand on activated endothelial cells. Many groups have linked Notch3 expression to clinical prognosis in advanced ovarian cancer. Jung et al, observed elevated levels of Notch3, Jagged1 and Jagged2 in serous ovarian cancer samples as compared to benign controls [35]. High Notch3 expression correlated significantly with worse overall survival and clinical chemoresistance.

Today antiangiogenic therapy based on anti-VEGF bevacizumab is used as a maintenance therapy in advanced ovarian cancer. While such treatment can target vessel formation, we show here a non-perfusion dependent role for endothelial cells through Notch signaling. To date, early clinical trials have provided little data regarding the efficacy of GSIs in ovarian cancer patients. A recent phase I clinical trial using the GSI RO4929097 in a range of advanced solid tumors reported prolonged stable disease in three of nine ovarian cancer patients. While other studies were not conclusive, there might be a place for Notch inhibition in advanced ovarian cancer in combination with other therapeutic strategies as we move toward personalized and precision medicine. Indeed, the genetic characterization of tumors could potentially identify a subset of tumors with aberrant Notch signaling that would constitute an ideal target for specific inhibitors.

## Conclusions

As mentioned previously, we have illustrated in several studies the role of angiocrine factors in tissue homeostasis and in several cancer models. Here, once again, our study points out this role in ovarian cancer resistance and might shed light on mechanisms pertaining to residual disease. If confirmed in translational study targeting such interaction could lead to better disease control and lower recurrence rate.

## Abbreviations

OCC: ovarian cancer cells
EC: endothelial cells
E4^+^ECs: Akt-activated endothelial cells transfected with E4ORF gene
TM: tumor microenvironment

## Availability of data and materials

The datasets used and/or analysed during the current study are available from the corresponding author on reasonable request.
